# The Student Loan Trap

**DOI:** 10.1120/jacmp.v16i5.6007

**Published:** 2015-08-19

I would like for you to join me in a thought exercise to explore how and why our current generation of students are compelled to shoulder up to $100,000 in graduate school debt to enter our profession. Not only are the debt levels onerous, these loans typically bear interest rates of up to 7%. In contrast, it is easy to get a car loan for around 4%. What is the meaning of this for our newly minted generation of professional colleagues?

I remember a time when tuition was affordable and it was relatively easy to complete a master's or doctorate in medical physics without any debt at all. That was only a generation ago. What changed and why? If we look across the pond, nations of the European Union seem to be working to eliminate university tuition. Germany did this in 2014 (http://www.slate.com/blogs/browbeat/2014/10/10/germany_college_is_free_there_even_for_foreign_students_why.html).

Yes, I believe a good education is worth paying for, and maybe free tuition is an unreasonable goal in the United States. However many US universities seem to be swimming in gifts, grants, endowments, and other resources. Why are some graduate medical physics program tuitions so high, and why have we been so willing and unconcerned to see these tuitions rise so dramatically? Why have tuitions increased at double‐digit rates beyond inflation? Is it because the universities were able to do so because students had access to virtually unlimited amounts of funds in the form of loans guaranteed by the Federal Government (http://trends.collegeboard.org/college‐pricing/figures‐tables/tuition‐fees‐room‐board‐time‐1974‐75‐2014‐15‐selected‐years)?

The numbers are not benign. People in the United States have accumulated over $1.2 trillion in student debt, surpassing credit card debt and automobile loan debt. Also, student debt is unlike any other type of debt. It is the only debt that cannot be discharged, even in bankruptcy. Virtually any other kind of debt — including medical bills, mortgage, credit cards and car loans, even gambling losses — can be discharged in bankruptcy, allowing the unfortunate a chance to restore their footing through an arduous restructuring overseen by a court. How did we get to this place? Here are some landmarks:

- 1965 — As part of his ‘Great Society’, Lyndon Johnson signs the *Higher Education Act*. Students are now able to afford college with federally guaranteed loans.
- 1978 — Stories surfaced of some graduates discharging their student loan debt by filing for bankruptcy immediately after graduation. The *Bankruptcy Reform Act* disallowed for discharging of student loans for five years after the first loan payment.
- 1990 — The non‐discharge period was extended to seven years.
- 1998 — Congress completely eliminated the ability to discharge federal student loan debt in bankruptcy.
- 2005 — Congress provided non‐discharge protection to private student loan lenders. Now, government and private student loans are essentially impossible to discharge.

Additionally, the following protections were removed from student loans:

- statute of limitations on collections
- *Truth in Lending Act*
- *Fair Debt Collection Practices Act*
- the right to refinance
- adherence to state usury laws

Harsh collection methods are reserved for student loans. Miss a few payments and you may experience (http://www.collegescholarships.org/research/student‐loans/):

- wage garnishment without a court order
- suspension of state professional licenses
- garnishment of social security/disability income
- withholding IRS tax refunds

Today, one out of every six Americans who owes money on a student loan is in default. There are about 6 million Americans who are at least 12 months behind on their student loan payments. Only 39% are paying down balances.

So why are things the way they are? Who benefits from this situation? One reality is that the US Federal Government profits from about $50 billion per year from student loan debt (http://www.usatoday.com/story/news/2013/06/16/us‐government‐projected‐to‐make‐record‐50b‐in‐student‐loan‐profit/2427443/). Does it, therefore, make sense for our system to penalize the youngest and most vulnerable of our colleagues trying to make it in a difficult economy?

I do not expect anything to happen respecting student debt relief. Why? Because America's higher learning system itself is dependent on perpetuating the status quo. Because if loans are harder to come by, college tuitions would be subject to downward pressure and the business model of the modern university would be threatened. Even if it makes little sense for the Federal Government to profit at the expense of graduate student debt, the Universities need the revenue from these artificially high tuitions (http://www.forbes.com/sites/jeffreydorfman/2014/04/19/student‐loan‐profits‐show‐government‐should‐get‐out‐of‐student‐loan‐business/).

My counsel to anyone considering medical physics as a career is to identify a graduate program that has low tuition and a good record for placing its students in residency programs. Make every effort to qualify for in‐state tuition. Embrace the reality that some program graduates will not successfully match to enter a residency program (http://www.campep.org/2014AnnualResidencyReport.pdf). Work hard, get your Board Certification, and pay off your loans as soon as you possibly can. Live frugally and save. And consider your salary could be about the same as a starting general practice physician, many of whom have up to $200,000 and more in student loans to pay off. Medical physics can still be a smart career choice, if you do it right.

Michael D. Mills, PhD

Editor‐in‐Chief

